# Dynamic contrast enhanced MRI reveals impaired blood brain barrier in basal forebrain region in patients with Alzheimers disease: A pilot study

**DOI:** 10.1002/alz.089915

**Published:** 2025-01-09

**Authors:** Ondrej Lerch, David Kala, Zuzana Nedelska, Haris Hadzic, Jakub Otahal, Jakub Hort

**Affiliations:** ^1^ Charles University, Second Faculty of Medicine and Motol University Hospital, Prague Czech Republic; ^2^ International Neurodegenerative Disease Research Center, Prague, Prague Czech Republic

## Abstract

**Background:**

Blood brain barrier (BBB) is a protective layer of cells that separates the circulatory system from the brain. Its dysfunction is one of the possible mechanisms leading to onset of Alzheimer's disease (AD), a progressive neurodegenerative disease and a leading cause of dementia worldwide. Dynamic contrast enhanced magnetic resonance imaging (DCE‐MRI) is a imaging technique allowing regional assessment of BBB breakdown by estimating local metrics of capillary permeability such as K‐trans (volume transfer constant). We used DCE‐MRI to examine BBB dysfunction in regions affected early in the course of AD ‐ hippocampus, entorhinal cortex (EC) and basal forebrain (BF) nuclei.

**Method:**

A pilot group of 29 participants ‐ 17 cognitively normal, 12 with mild cognitive impairment (MCI) due to AD from Czech Brain Aging Study underwent DCE‐MRI. K‐trans maps were estimated using ROCKETSHIP software implemented within MATLAB 2023b. Segmentations of hippocampal head, body and tail, anterolateral and posteromedial EC and BF nuclei were obtained using in house developed pipeline. Average K‐trans values were extracted for each region. Regional differences in BBB permeability between groups were assessed using ANOVA.

**Result:**

Participants in the MCI group had increased mean K‐trans in anterior (K‐trans_CN_= 0.752 x10^‐3^min^‐1^; K‐trans_MCI_= 0.991 x10^‐3^min^‐1^, p = 0.023) and posterior (K‐trans_CN_ = 0.656 x10^‐3^min^‐1^; K‐trans_MCI_ = 0.984 x10^‐3^min^‐1^, p = 0.018) part of nucleus basalis Meynerti (NBM). There were no other significant differences between regional BBB permeability in measures structures (p>0.05)

**Conclusion:**

Our pilot data show regional BBB dysfunction in NBM region in patients with MCI due to AD. This may imply regional BBB vulnerability of BF or another ongoing process such as hyperactivation or inflammation, suggesting a link between BBB dysfunction and early pathological changes in the BF area. Verification of these results on larger dataset is necessary before any further conclusions can be made.

